# The effectiveness of a brief intervention for intensive care unit patients with hazardous alcohol use: a randomized controlled trial

**DOI:** 10.1186/s13054-024-04925-z

**Published:** 2024-04-30

**Authors:** Eliisa Nissilä, Marja Hynninen, Ville Jalkanen, Anne Kuitunen, Minna Bäcklund, Outi Inkinen, Johanna Hästbacka

**Affiliations:** 1 Department of Perioperative and Intensive Care Medicine, Intensive Care Units, Helsinki University Hospital and Helsinki University, Haartmaninkatu 2, PL 340, 00029 Helsinki, Finland; 2 Department of Intensive Care, Tampere University Hospital, Wellbeing District of Pirkanmaa and Tampere University, Tampere, Finland; 3 Perioperative Services, Intensive Care and Pain Medicine, Turku University Hospital, Wellbeing District of Southwest Finland and University of Turku, Turku, Finland

**Keywords:** Hazardous alcohol consumption, Intensive care unit, Critical care, Brief intervention, Mortality, AUDIT-C, RCT

## Abstract

**Background:**

Screening for hazardous alcohol use and performing brief interventions (BIs) are recommended to reduce alcohol-related negative health consequences. We aimed to compare the effectiveness (defined as an at least 10% absolute difference) of BI with usual care in reducing alcohol intake in intensive care unit survivors with history of hazardous alcohol use.

**Methods:**

We used Alcohol Use Disorder Identification Test-Consumption (AUDIT-C) score to assess history of alcohol use.

*Patients:* Emergency admitted adult ICU patients in three Finnish university hospitals, with an AUDIT-C score > 5 (women), or > 6 (men). We randomized consenting eligible patients to receive a BI or treatment as usual (TAU).

*Intervention*: BI was delivered by the time of ICU discharge or shortly thereafter in the hospital ward.

*Controls*: Control patients received TAU.

*Outcome*: The primary outcome was self-reported alcohol consumption during the preceding week 6 and 12 months after randomization. Secondary outcomes were the change in AUDIT-C scores from baseline to 6 and 12 months, health-related quality of life, and mortality. The trial was terminated early due to slow recruitment during the pandemic.

**Results:**

We randomized 234 patients to receive BI (N = 117) or TAU (N = 117). At 6 months, the median alcohol intake in the BI and TAU groups were 6.5 g (interquartile range [IQR] 0–141) and 0 g (0–72), respectively (*p* = 0.544). At 12 months, it was 24 g (0–146) and 0 g (0–96) in the BI and TAU groups, respectively (*p* = 0.157). Median change in AUDIT-C from baseline to 6 months was − 1 (− 4 to 0) and 2 (− 6 to 0), (*p* = 0.144) in the BI and TAU groups, and to 12 months − 3 (− 5 to − 1) and − 4 (− 7 to − 1), respectively (*p* = 0.187). In total, 4% (n = 5) of patients in the BI group and 11% (n = 13) of patients in the TAU group were abstinent at 6 months, and 10% (n = 12) and 15% (n = 17), respectively, at 12 months. No between-groups difference in mortality emerged.

**Conclusion:**

As underpowered, our study cannot reject or confirm the hypothesis that a single BI early after critical illness is effective in reducing the amount of alcohol consumed compared to TAU. However, a considerable number in both groups reduced their alcohol consumption.

*Trial registration:* ClinicalTrials.gov (NCT03047577).

**Supplementary Information:**

The online version contains supplementary material available at 10.1186/s13054-024-04925-z.

## Background

Hazardous alcohol use is an important contributing factor to morbidity and mortality worldwide, with social- and economic costs [1–3]. According to studies using screening questionnaires, 21–34% of patients treated in intensive care units (ICUs) reported a history of hazardous alcohol use [4–8]. A history of hazardous alcohol use is thus common among patients admitted to the ICU [4–9].

Screening for alcohol use and performing brief interventions (BI) to motivate patients to reduce hazardous alcohol consumption have been reported to be effective strategies in primary health care and hospital settings [10–15] Patients with a history of hazardous alcohol use may be more receptive to learning about the risks of alcohol use during hospitalization [16, 17]. The first step in the treatment of patients suspected of hazardous alcohol consumption is screening, typically using validated questionnaires. A positive screening result leads to a BI which usually consists of six key elements: feedback, responsibility, advice, menu for change, empathy and enhancing self-efficacy (summarized by the acronym FRAMES) [10, 18]. In addition, the BI may include provision of self-help materials and contact information on additional support available [19].

The period after a severe illness may be a teachable moment, suitable for providing information and support and for motivating patients to reduce their alcohol use. In this study, we aimed to examine the effectiveness of a BI compared to treatment as usual (TAU) in reducing hazardous alcohol use in ICU survivors. Our objective was to compare the alcohol intake between patients receiving BI with those with TAU, at 6 and 12 months after randomization. We hypothesized that BI would lead to decreased alcohol consumption compared to TAU, 6 and 12 months after randomization.

We also compared the change in AUDIT-C scores from baseline, health-related quality of life (HRQoL), and survival at 12 months.

## Methods

### Trial design

This was a randomized controlled parallel group trial with a 1:1 group allocation. The study protocol has been registered at ClinicalTrials.gov (NCT03047577).

### Participants

Participants were patients with a history of hazardous alcohol consumption, admitted to the ICUs of three university hospitals (Helsinki, Tampere, and Turku). For identifying potentially eligible patients, we used the Alcohol Use Disorders Identification Test-Consumption (AUDIT-C), which is a short three-item version of the Alcohol Use Disorders Identification Test (AUDIT) and includes questions about alcohol consumption [20, 21]. AUDIT-C is used in Finnish ICUs for assessing alcohol consumption. It is obtained from patients or a close family member by clinical staff and recorded in the patient data management system (PDMS) as a part of a comprehensive health evaluation. Study coordinators screened the PDMS AUDIT-C scores and informed the physicians in the research team of a patients potentially eligible for assessment. Inclusion criteria were a history of hazardous alcohol use within the preceding year (AUDIT-C score > 5 for women, > 6 for men) [22–24], age ≥ 18 years, and emergency admission to the ICU. Exclusion criteria were terminal illness, transition to palliative care, diagnosed with a major psychiatric comorbidity, known impaired cognitive functioning, diagnosis of a memory disorder, impaired level of consciousness on discharge from the ICU, drug addiction, ongoing treatment for alcohol addiction before ICU admission, insufficient language skills to communicate in the local language (Finnish or Swedish), and a high probability of being lost to follow-up (no permanent address or telephone number). For patients fulfilling the inclusion and none of the exclusion criteria, we asked for consent to participate in the study when the patients had regained their capacity for decision making (shortly before ICU discharge or during post-ICU care in the hospital ward). After written consent, all patients completed an AUDIT questionnaire. We then randomized the patients to a BI or TAU.

### Intervention

The BI was delivered in the ICU or after ICU discharge in the hospital ward in a single, face-to-face session by a member of the research group (ICU nurses or physicians) trained to conduct BIs. Training for conducting BIs was provided by A-Clinic Foundation, Helsinki, Finland. The BI included motivational interviewing techniques and feedback about the patients´ recent alcohol consumption habits and information on health-related risks of hazardous alcohol use [19]. The baseline AUDIT questionnaire was used as the basis for the discussion. During the intervention, we asked the patients about their willingness to change their alcohol use behavior, and how confident they were in their ability to change, with both questions answered on a 1–10 scale (1 representing the lowest and 10 the highest possible willingness and confidence in their ability to change). End of the BI focused on support and advice to reduce alcohol use [19, 25]. BI also included providing written material and contact details for organizations providing support in the patient´s district of residence. In addition, the BI included an option to speak with a social worker in the hospital. We did not measure the duration of the intervention.

The control group was assigned to TAU, with no interventions, other than completing the AUDIT questionnaire before randomization. They were free to seek support independently, and to engage in eventual discussions with hospital personnel in post-ICU care about alcohol use interventions.

### Outcomes and measures

The primary outcome measures were self-reported alcohol consumption during the preceding week (converted later to 100% ethanol in grams by the research team, see Additional file 1 for details) 6 and 12 months after randomization. The patients were allowed to choose follow-up either by a mailed questionnaire or structured telephone interview. We made two attempts to contact the participants in case of no response at the first attempt. The structured telephone interview and questionnaire at the 12-month follow-up were identical and included AUDIT questionnaire and the health-related quality of life questionnaire EQ-5D-3L [26], in addition to the question about alcohol consumption during the preceding week (see electronic Additional file 1 for a detailed description of the interview). Secondary endpoints were the change in AUDIT-C from baseline to 6 and 12 months (derived from AUDIT questions 1–3 = AUDIT-C) after randomization, HRQoL assessed with EQ-5D-3L questionnaire, and mortality at 12-months. The follow-up interviews in both groups included questions about the participants´ current willingness and confidence in their ability to change their alcohol use habits.

We collected following data in an electronic case report system (Absolute Imaginary Software Ltd., Kauniainen, Finland) from hospital electronic patient records (PICIS 8.6: Care Suite, USA; Uranus 8.4.6.16: CGI, Canada; Apotti: Epic systems, USA); Age, sex, type of housing, ICU admission diagnosis, simplified acute physiology score II (SAPS II) [27], sequential organ failure assessment score (SOFA) [28] from first 24 h of ICU stay, length of stay in ICU (ICU LOS). We calculated Charlson comorbidity index (CCI) without age points based on diagnoses from the patient records.

### Sample size

According to the original sample size calculation, we planned to recruit 600 patients, accounting for an estimated 25% attrition rate, estimated on basis of literature on BI in hospitalized patients [10]. We conducted a power analysis based on a two-tailed test, with α = 0.05 and 0.80 power to detect a 10% difference in the primary outcome between groups. The chosen difference was judged clinically significant [29] and was based on the result in a systematic review on BI research in emergency settings [12]. We planned an interim analysis after the 12-month-follow-up data of 200 patients would be collected, but as the COVID-19 pandemic significantly slowed down recruitment (see Additional file 1: Figure E1), we decided to stop recruiting at that point. The final number of recruited patients was 234 (39% of the calculated sample size).

**Fig. 1:**
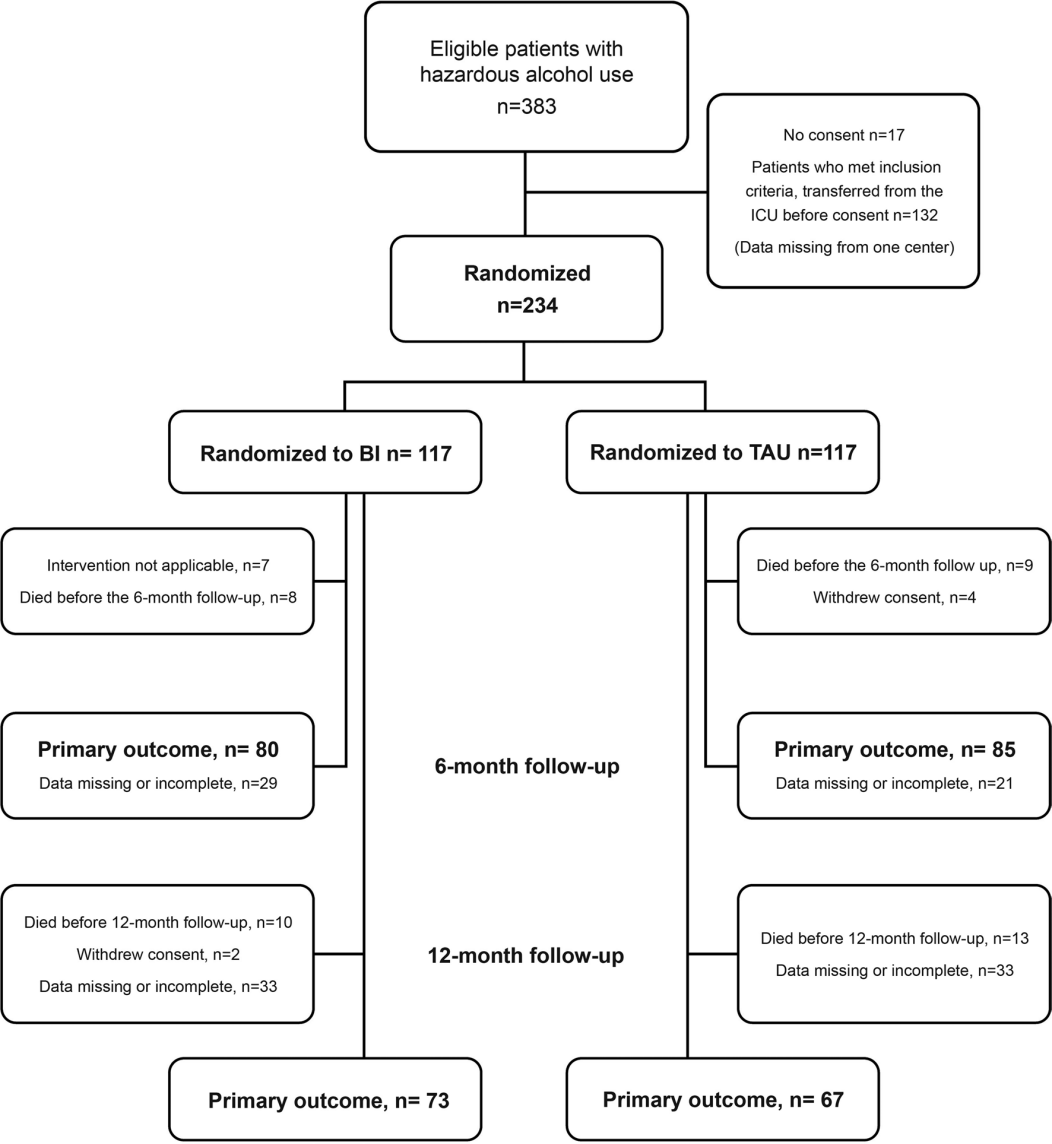
Flow chart showing the number of patients randomized to the brief intervention (BI) or treatment as usual (TAU). Number of patients with available follow-up data, withdrawals, and non-survivors are shown. We replaced missing values concerning the primary outcome by carrying forward the values of the last observations

### Randomization

We randomized patients to either the BI or TAU with 1:1 group allocation using a centralized computer-based randomization sequence in permuted blocks with a block size varying from 2 to 6. We used an unbiased Fisher-Yates (Durstenfeld) algorithm to shuffle the blocks (Absolute Imaginary Software Ltd., Kauniainen, Finland). We used sex and alcohol addiction, as defined by a score of ≥ 20 points (out of 40) in the baseline AUDIT [30] or a score of ≥ 1 points in AUDIT questions 4–6 indicating dependency [20], and center as stratification variables to minimize between-group differences in variables that could affect the primary outcome.

### Blinding

Blinding was not feasible due to the way in which the BI was delivered. However, the outcome assessors performing the follow-up interviews were blinded to the group allocation. To ensure blinding, we instructed the assessors not to ask the patients about their group allocation**.**

### Statistical analysis

Continuous variables are presented as medians and interquartile ranges (IQRs), and categorical data as absolute numbers and percentages. We performed analyses of alcohol consumption during the preceding week and change in AUDIT-C score from baseline to 6 and 12 months after randomization in the intention-to-treat (ITT) population and, secondarily, in the per protocol (PP) population. We replaced missing values concerning the primary outcome and AUDIT-C scores by carrying forward the values of last observations. For comparisons of the distributions of non-normally distributed data, we used the nonparametric Mann–Whitney* U* test. For comparison of categorical data, we used the chi-square test. We calculated hazard ratios (HRs) with 95% confidence intervals (95% CIs) for 12-month mortality using Cox regression analysis, for crude mortality, and adjusting for age, SAPS II, and CCI without age points. We performed no interim analysis. We considered a *p*-value of < 0.05 as statistically significant. We used IBM SPSS Statistics for Windows, Version 27.0. (IBM Corp., Armonk NY, USA) and Jamovi project® (version 2.4.14) for the analyses. We follow CONSORT 2010 statement for reporting of this trial [31].

### Ethics approval and consent to participate

The Ethics committee of Helsinki University Hospital approved the protocol (HUS/2046/2016), and permission to perform the study was granted by local authority of each participating hospital. The patients were treated according to the principles in Declaration of Helsinki and its later amendments. All patients provided a written informed consent for participation.

## Results

The trial recruited between March 21st 2017, and July 12th, 2021, and the follow-up of the last patient was conducted July 12th, 2022.

### Participants

The study flow chart, including randomization of the patients, numbers of patients with available follow-up information, non-survivors, and withdrawal are presented in Fig. 1. Follow-up information was missing in 23% of cases at 6-month follow-up and in 32% of cases at the 12-month follow up. The follow-up data were incomplete. Information on the missing data is provided in Additional file 1: Table E1. Seven patients assigned to the BI group did not receive the intervention due to transfer to another hospital before the intervention, or impaired medical status.

Table 1 presents the characteristics of all patients. The baseline characteristics were balanced between the groups considering sex, age, and number of patients with alcohol dependency (Table 1). Additional file 1: Table E2 shows the main ICU admission diagnoses.

**Table 1:** Characteristics of the patients in the BI and treatment as usual (TAU) groups

	BI n = 117	TAU n = 117
Age, years, median (IQR)	54 (47–64)	56 (48–64)
Sex male, n (%)	94 (80.3)	96 (82.1)
Housing type		
Home, alone/single	59 (50.9)	54 (46.6)
Home, with other/spouse	50 (43.1)	54 (46.6)
Dormitory	1 (0.9)	2 (1.7)
Sheltered accommodation	1 (0.9)	0
Other	5 (4.3)	6 (5.2)
Inclusion AUDIT-C score, median (IQR)	8 (7–10)	9 (8–11)
Baseline AUDIT, median (IQR)	18 (12–23)	19 (12–24)
Alcohol addiction^ψ^, n (%)	79 (67.5)	77 (65.8)
Surgical admission, n (%)	20 (17.5)	28 (24.6)
ICU LOS, days, median (IQR)	5 (3–8)	5 (3–9)
SAPS II, median (IQR)	31 (22–41)	29 (20–36)
SOFA 24, median (IQR)	7 (4–9)	7 (5–9)

IQR, interquartile range; ICU LOS, Intensive Care Unit Length of Stay**;** SAPS II, Simplified acute physiology score II; SOFA 24, Sequential organ failure assessment score during the first hours in the ICU. ^ψ^Alcohol addiction was defined by ≥ 20 points (out of 40) in the baseline Alcohol Use Disorder Identification Test (AUDIT), or a score of 1 or more points in AUDIT questions 4–6

### Alcohol consumption during the preceding week at the 6- and 12-month follow-ups

**Fig. 2:**
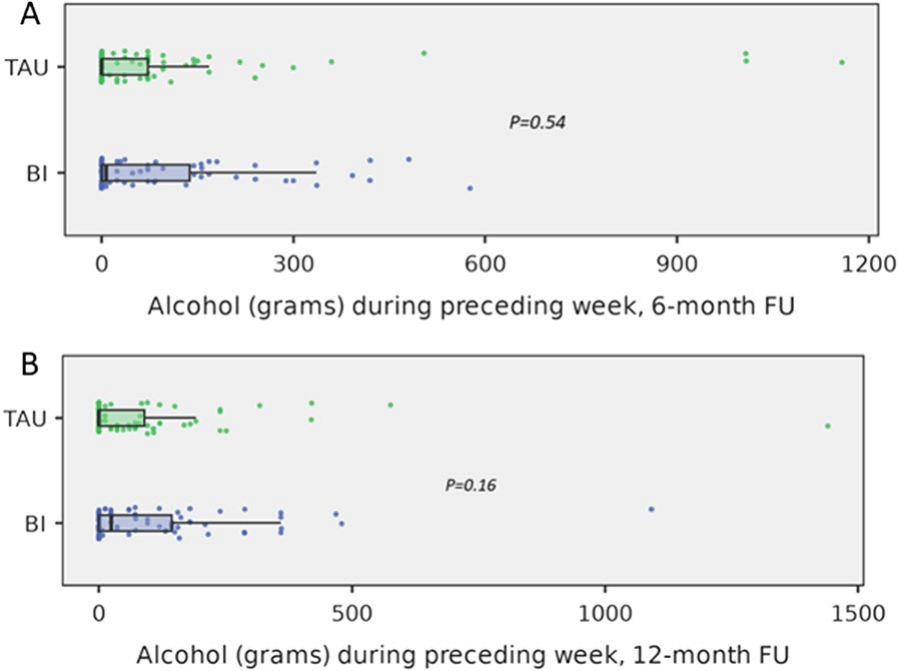
**A** and** B** Alcohol consumption in BI and TAU groups during the preceding week in grams of pure ethanol, at six (2A) and twelve (2B) follow-ups

**Fig. 3:**
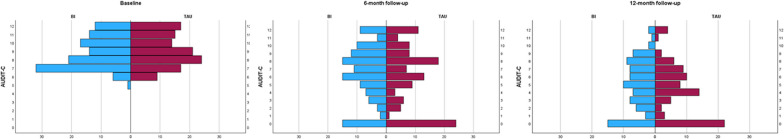
Distribution of AUDIT-C scores in BI and TAU groups at baseline, 6-and 12-month follow-ups

Table 2 presents between-group comparisons of the amount of alcohol consumption at 6 and 12 months after randomization in the intention-to-treat (ITT) population. The distribution of intake in each group is depicted in Fig. 2. and according to sex and group in Additional file 1: Figure E2A and E2B. Figure 3 shows distribution of AUDIT-C scores in BI and TAU groups at baseline, 6-and 12-month follow-ups. Patients who died or withdrew consent before the end of the follow-ups were not included in analysis. Thirty-nine (36%) patients in the BI group and 44 (42%) patients in the TAU group reported no alcohol use during the preceding week at the 6-month follow-up (*p* = 0.532). At the 12-month follow-up, 34 (32%) patients in the BI group and 35 (35%) patients in the TAU group reported no alcohol use during the preceding week (*p* = 0.495). Additional file 1: Table E3 presents the results of between-group comparisons of the 6- and 12-month alcohol consumption, AUDIT-C scores, and change in AUDIT-C scores in the PP populations.

**Table 2:** Comparisons of the amount of alcohol intake during the preceding week converted to grams (g) of pure ethanol, AUDIT-C scores and change in AUDIT-C scores (ΔAUDIT-C) 6 and 12 months after randomization in the brief intervention (BI) group and treatment as usual (TAU) groups

	BI n = 117	TAU n = 117	*p*-value
*6-month follow-up*			
Alcohol consumption (g), median (IQR)	6.5 (0–141) n = 80	0 (0–72) n = 85	0.544
AUDIT-C, median (IQR)	6 (4–9) n = 109	6 (2–8) n = 104	0.548
ΔAUDIT-C score, median (IQR)	− 1 (− 4 to 0) n = 109	− 2 (− 6 to 0) n = 104	0.144
Abstinent: AUDIT score = 0, n (%)	5 (4) n = 117	13 (11) n = 117	0.052
Hazardous alcohol use according to WHO limits^β^, n (%)	12 (10.3)	8 (6.8)	0.260
Hazardous alcohol use according to AUDIT-C, n (%)^γ^	75 (64.1)	69 (59.0)	0.420
*12-month follow-up*			
Alcohol consumption (g), median (IQR)	24 (0–146) n = 86	0 (0–96) n = 83	0.157
AUDIT score, median (IQR)	8 (4–14) n = 84	6 (0–10) n = 82	0.054
AUDIT-C, median (IQR)	5 (2–8) n = 84	4 (0–7) n = 82	0.243
ΔAUDIT-C score, median (IQR)	− 3 (− 5 to − 1) n = 84	− 4 (− 7 to − 1) n = 82	0.187
Abstinent: AUDIT score = 0, n (%)	12 (10) n = 117	17 (15) n = 117	0.195
Hazardous alcohol use according to WHO limits^β^, n (%)	13 (11.1)	6 (5.1)	0.105
Hazardous alcohol use according to AUDIT-C, n (%)^γ^	37 (31.6)	32 (27.4)	0.437

Categorical data are presented as percentages and absolute numbers (n), continuous variables are expressed as medians and interquartile ranges (IQR). Alcohol consumption is presented as converted to grams (g) of pure ethanol. ΔAUDIT-C is the change in AUDIT-C scores from the baseline scores to follow-up scores; IQR, interquartile range; AUDIT-C, Alcohol Use Disorder Identification Test Consumption., ^β^Self-reported alcohol intake during preceding week using WHO limits for hazardous use of alcohol: 140 g pure ethanol per week in women and 280 g pure ethanol per week in men; ^γ^AUDIT-C > 5 in women, AUDIT-C > 6 in men

**Table 3:** Results of Cox proportional hazard analysis of 12-month mortality, adjusted for age, SAPS II (representing severity of acute disease), and Charlson comorbidity index (CCI)

	HR (95% CI)
Age	1.009 (0.972–1.1048)
SAPS II	0.984 (0.953–1.1017)
CCI	1.516 (1.206–1.905)*
BI	0.714 (0.311–1.637)

HR, hazard ratio; CI, confidence interval; SAPS II, Simplified acute physiology score II without age score; CCI, Charlson comorbidity index without age points; BI, brief intervention

*A statistically significant result

### Willingness to change and confidence in ability to change alcohol use habits

The correlation coefficients for the 12-month ΔAUDIT-C score with willingness and confidence in ability to change reported at the 6-month follow-up were − 0.325 (n = 73, *p* = 0.005) and − 0.306 (n = 73, *p* = 0.008) in BI group and − 0.345 (n = 77, *p* = 0.002) and − 0.379 (n = 78, *p* < 0.001) in the control group, respectively. There were no differences between the BI and control groups in either willingness to change alcohol use habits or confidence in their ability to change (scale: 1–10) at the 6- and 12-month follow-ups (Additional file 1: Table E5).

### Quality of life at the 12-month follow-up

Patients in the BI group reported fewer problems with daily self-care than the patients in the TAU group. Otherwise, the quality-of-life survey results did not differ between the groups. Additional file: Table E6 shows the EQ-5D-3L results at the 12-month follow-up. There was no difference between BI and TAU groups in general health (median: 80.0 [53.8–86.0] and 80.0 [58.0–85.0], respectively) on a 0–100 scale (*p* = 0.74).

### Mortality

Patients who withdrew consent were not included in the mortality analyses, except one patient, who approved the use of survival data. Mortality at 12 months was 9% (10/114) in the BI group and 11% (13/114) in the TAU group (*p* = 0.509). The HR for crude 12-month mortality was 0.716 (95% CI 0.318–1.612). In Cox regression analysis adjusting for age, SAPS II without age points and CCI without age points, only CCI was independently associated with 12-month mortality (Table 3). The Baseline AUDIT scores of the 12-month non-survivors (median: 23, IQR 22–29), were higher than those of the 12-month survivors (median: 17, IQR 11–23) (*p* = 0.002).

## Discussion

We conducted a randomized, controlled multi-center study to assess whether a BI at the time of ICU discharge affected alcohol consumption 6 and 12 months later in patients with a history of hazardous alcohol use. To the best of our knowledge, this is the first randomized controlled trial to examine the effect of a BI on alcohol consumption after critical illness, and the first study to attempt to study the effectiveness of a BI in general ICU patients with a history of hazardous alcohol consumption. Because the trial was terminated early it lacks statistical power to reject or confirm the hypothesis of a 10% reduction in alcohol consumption with a BI delivered early after ICU discharge, compared to treatment as usual. On the other hand, no trend was observed that the BI would have decreased the alcohol consumption more than TAU. An important finding of our study was that regardless of the group allocation, considerable number of participants in each group reported no use of alcohol during the week preceding the 6- and 12-month follow-up assessments. Six months after randomization, 4% of individuals in the BI group and 11% of those in the TAU group reported total abstinence. At the 12-month follow-up, the corresponding proportions were 10% and 15% in the BI and TAU groups, respectively. Surprisingly, the prevalence of reported abstinence had increased in both groups at the 12-month follow up despite two-thirds of the participants being alcohol dependent at baseline. Screening for hazardous alcohol consumption and BIs are recommended in all healthcare encounters by WHO and Finnish Current Practice Guidelines [19, 32] and ICU survivors should receive support in reducing their alcohol consumption to avoid further negative health consequences. Our results suggest that critical illness is a strong motivational factor for reducing alcohol consumption.

The effectiveness of BIs has been demonstrated in primary care settings [11, 13, 33]. There is conflicting evidence on the effectiveness of BIs in emergency settings [12, 34], with some studies finding that BIs were not effective in emergency settings [35, 36], but another study finding that alcohol-related injury may increase the effectiveness of BI [37]. The usefulness of BIs may depend on the target population. For example, BIs in emergency settings were found not to be useful for patients with substance abuse, admitted because of violence-related events [36]. However, alcohol interventions to trauma patients were associated with reduced alcohol-related injuries [38, 39]. The results of studies on the effectiveness of BIs for hazardous alcohol use in hospitalized patients are conflicting [10, 12, 39–42]. We observed no trend to more decreased alcohol consumption after BI than TAU, but AUDIT-C scores decreased significantly in both the BI and TAU groups. The BI was delivered in the ICU or shortly after ICU discharge in the hospital ward. The timing was soon after critical illness and perhaps not optimal for a BI. Possibly, the experience of critical illness, participating in the study, and answering AUDIT questionnaire rather than the BI may have motivated the patients (in both groups) to reduce hazardous alcohol use. According to the World Health Organization (WHO), hazardous alcohol use is defined as over 20 g per day for women, and over 40 g per day for men [43]. In our study, only a few patients reported consuming more alcohol than the WHO recommendation at the 6- and 12-months follow-ups, but according to AUDIT-C scores from the same time point hazardous use was still prevalent. This discrepancy is likely explained by irregular drinking, a common drinking pattern in Finland [44].

A desire to change drinking habits is thought to be the main component of a successful BI [45]. Willingness to change is an important part of motivation and readiness to change [45]. We found no difference between the groups in their willingness to change alcohol use habits or the participants´ confidence in their ability to change, at the follow-up assessments. A previous study reported that willingness and motivation to change alcohol use habits were associated with successful treatment outcomes for hazardous alcohol use [46–48]. In addition, the confidence in one’s ability to change is important in reducing harmful alcohol consumption [45]. In our study, the participants’ confidence in their ability to change was high in both groups at both follow-ups. In both groups, we found a moderate correlation between both willingness to change and confidence in the ability to change at the 6-month follow-up and the change in AUDIT-C scores from 6 to 12 months.

Although 12-month mortality was low in our population, the baseline AUDIT score was higher in non-survivors than survivors. We found no difference in crude or adjusted mortality between the intervention and control groups. This is contrary to the results of a previous meta-analysis, where a BI associated with decreased mortality [10, 15]. In our study, there were no major between-group differences in the quality of life at the 12-month follow-up although the patients in the BI group reported coping self-care better than those in the control group.

Our study has several strengths. It was a multicenter study and included both emergency medical and surgical patients’ admissions, which increases the generalizability of our results. We used central randomization, with stratification for center, sex, and dependency, and delivered the intervention in a structured manner using trained investigators and recommended interview techniques. In addition, the outcome assessors were blinded to the group allocation.

Our study has limitations. Patient recruitment was terminated early due to the slow recruitment during the pandemic. As a result, we recruited only 39% of the calculated sample size. The loss to follow-up rate was higher than the estimated 25%, which is a recognized source of bias in studies in similar populations [10–12]. We did not analyze the results separately in women and men. Due to the nature of the intervention, blinding was not feasible in delivering the intervention. Although not routinely available, patients in both groups may have received support for reducing alcohol consumption, both during and after hospitalization. AUDIT interview at baseline with both groups (for stratification purposes) may be perceived as a type of support. The primary endpoint was based on self-report, involving the inherent weaknesses of self-report, and risk of response and recall bias. Our primary endpoint probably reflects short-term alcohol consumption, especially in subjects with alcohol addiction, supported by our finding of different prevalence of hazardous use when using the AUDIT-C and recent intake as methods of assessment. Both belong, however, to the recently published core outcome set for BI trials [49]. The collection of data for the primary endpoint using two alternative methods may have caused bias, and despite efforts there were missing data. Finally, we cannot exclude selection bias regarding the screening process not covering consecutive patients, patients´ willingness to participate, and the response rate (e.g., favoring participation of motivated patients and responses from patients with successful reduction of alcohol use).

Despite these limitations, our results are important. Reducing hazardous alcohol consumption is crucial to decrease the negative health consequences associated with hazardous alcohol use. Studies focusing on optimal timing, number and type of intervention, and objective method for screening and assessment of outcome in ICU survivors with history of hazardous alcohol use are called for. A follow-up clinic two to three months after hospital discharge might be a suitable setting for a BI intervention trial, as our results point out that the ICU survivors are motivated to change their alcohol use habits. In future studies, the sample size calculation should consider a higher attrition rate and make all efforts to reduce missing data. Outcomes collected should align with the published core outcome set [45].

## Conclusion

Because of early termination of the trial, the results of this randomized controlled study cannot confirm or reject the hypothesis that a BI early after ICU discharge leads to at least a 10% reduction in alcohol consumption compared to treatment as usual. Recommendations to screen for hazardous alcohol consumption and performing BIs are not abrogated by our results. On the contrary, the result that after critical illness a considerable number of patients decreased their alcohol use, and some became abstinent, suggests that critical illness is a strong motivating factor for changing behavior. Supporting this motivation may reduce the negative health consequences of hazardous alcohol consumption in this heterogeneous group of critically ill patients, but further research is needed to identify optimal methods.

### Supplementary Information

**Additional file 1: **Definitions, Description of the follow-up interview. **Figure E1.** Rate of recruitment. The first patients was randomized in Helsinki ICU at 31. March 2017, Tampere 13. August 2017 and Turku 7. May, 2018. Pandemic slowed down recruitment considerably. **Table E1.** Loss of follow-up information. Some of the follow-up information was returned but was filled incompletely. **Table E2.** ICU admission diagnosis categories. **Figure E2A.** Alcohol intake during preceding week in grams of pure ethanol according to sex. Comparison between patients randomized to BI and usual care at 6-month follow-up. **Figure E2B.** Alcohol intake during preceding week in grams of pure ethanol according to sex. Comparison between patients randomized to BI and usual care at 12- month follow-up. **Table E3.** Per protocol analysis: comparisons of the amount of alcohol consumption, AUDIT scores and change in AUDIT scores 6- and 12 months after randomization. **Table E4. **AUDIT-scores and subdomains at baseline, and at 6- and 12-month follow-up. **Table E5.** Willingness to change alcohol use habits (scale 1-10) and confidence in ability to change (scale 1-10) at baseline, and 6- and 12-month follow-up after ICU admission. **Table E6.** Reported EQ-5D-3L levels 12 months after ICU admission. CONSORT checklist.

## Data Availability

The written consent to participate in this study did not include public sharing of the data. Due to the sensitive nature of the research topic supporting data is not publicly available.

## Abbreviations

BI: Brief intervention
AUDIT-C: Alcohol use disorder identification test-consumption
ICU: Intensive care unit
AUDIT: Alcohol use disorder identification test
IQR: Interquartile range
SAPS II: Simplified acute physiology score II (SAPS II)
SOFA: Sequential organ failure assessment score
HR: Hazard ratio
CI: Confidence interval
LOS: Length of stay
CCI: Charlson comorbidity index
ITT: Intention-to-treat
PP: Per protocol
WHO: World Health Organization
