# Supplementary material for: The effectiveness of a brief intervention for intensive care unit patients with hazardous alcohol use: a randomized controlled trial
Source: Crit Care. 2024 Apr 30;28:145. doi: 10.1186/s13054-024-04925-z (PMC11061909; doi:10.1186/s13054-024-04925-z)
Supplement: Supplementary file 1 — Additional file 1: Definitions, Description of the follow-up interview. Figure E1. Rate of recruitment. The first patients was randomized in Helsinki ICU at 31. March 2017, Tampere 13. August 2017 and Turku 7. May, 2018. Pandemic slowed down recruitment considerably. Table E1. Loss of follow-up information. Some of the follow-up information was returned but was filled incompletely. Table E2. ICU admission diagnosis categories. Figure E2A. Alcohol intake during preceding week in grams of pure ethanol according to sex. Comparison between patients randomized to BI and usual care at 6-month follow-up. Figure E2B. Alcohol intake during preceding week in grams of pure ethanol according to sex. Comparison between patients randomized to BI and usual care at 12- month follow-up. Table E3. Per protocol analysis: comparisons of the amount of alcohol consumption, AUDIT scores and change in AUDIT scores 6- and 12 months after randomization. Table E4. AUDIT-scores and subdomains at baseline, and at 6- and 12-month follow-up. Table E5. Willingness to change alcohol use habits (scale 1-10) and confidence in ability to change (scale 1-10) at baseline, and 6- and 12-month follow-up after ICU admission. Table E6. Reported EQ-5D-3L levels 12 months after ICU admission. CONSORT checklist. [file 13054_2024_4925_MOESM1_ESM.docx]

**Additional File 1.**

The Effectiveness of a Brief Intervention for Intensive Care Unit Patients with Hazardous Alcohol Use: A Randomized Controlled Trial

Eliisa Nissilä, Marja Hynninen, Ville Jalkanen, Anne Kuitunen, Minna Bäcklund, Outi Inkinen, Johanna Hästbacka

**Contents**

[Definitions 1](#_Toc160449638)

[Description of the follow-up interview 2](#_Toc160449639)

[Figure E1. 4](#_Toc160449640)

[Table E1. 4](#_Toc160449641)

[Table E2. 5](#_Toc160449642)

[Figure E2A. 6](#_Toc160449643)

[Figure E2B. 6](#_Toc160449644)

[Table E3. 7](#_Toc160449645)

[Table E4 8](#_Toc160449646)

[Table E5. 9](#_Toc160449647)

[Table E6. 9](#_Toc160449648)

[CONSORT 2010 checklist 10](#_Toc160449649)

## Definitions

AUDIT-C: This questionnaire consists of the questions 1-3 of the AUDIT. We used AUDIT-C scores in screening hazardous alcohol consumption.

Hazardous alcohol consumption: In this study we considered hazardous alcohol consumption as AUDIT-C >5 fir women and >6. for men.

## Description of the follow-up interview

**Question 1. Please enter here the number of alcoholic beverages you consumed during the last week (7 days). Report as precisely as possible how many portions you have consumed.**

One standard drink in Finland is 12 grams of pure alcohol

Examples of one standard drink:

0,33 L can of medium-strength beer

12 cL glass of wine

4 cL of spirit drink

**Question 2. We kindly ask you to fill out the attached AUDIT questionnaire**

**Alcohol use disorders identification test (AUDIT) [38].**

The AUDIT is alcohol screen, which is scored on a scale of 0-40

Scoring: 0= 0 points, 1=1 point, 2= 2 points, 3=3 points, 4= 4 points.

0 to 7 indicates low risk

8 to 15 indicates increasing risk

16 to 19 indicates higher risk

20 or more indicates possible dependence

1. How often do you have a drink containing alcohol?
2. Never
3. Monthly or less
4. 2-4 times a month
5. 2-3 times a week
6. 4 or more times a week
7. How many units of alcohol do you drink on a typical day when you are drinking?
8. 1or 2
9. 3 or 4
10. 5 or 6
11. 7 or 9
12. 10 or more
13. How often have you taken 6 or more units (if female), or 8 or more (if male), on a single occasion in the last year?
14. Never
15. Less than monthly
16. Monthly
17. Weekly
18. Daily or almost daily
19. How often during the last year have you found that you were not able to stop drinking once you had started?
20. Never
21. Less than monthly
22. Monthly
23. Weekly
24. Daily or almost daily
25. How often during the last year have you failed to do what was normally expected from you because of your drinking?
26. Never
27. Less than monthly
28. Monthly
29. Weekly
30. Daily or almost daily
31. How often during the last year have you needed an alcoholic drink in the morning to get yourself going after a heavy drinking session?
32. Never
33. Less than monthly
34. Monthly
35. Weekly
36. Daily or almost daily
37. How often during the last year have you had a feeling of guilt or remorse after drinking?
38. Never
39. Less than monthly
40. Monthly
41. Weekly
42. Daily or almost daily
43. How often during the last year have you been unable to remember what happened the night before because you had been drinking?
44. Never
45. Less than monthly
46. Monthly
47. Weekly
48. Daily or almost daily
49. Have you or somebody else been injured as a result of your drinking?
50. No
51. Yes, but not in the last year
52. Yes, during the last year
53. Has a relative or friend, doctor or other health worker been concerned about your drinking or suggested that you cut down?

´0. No

´2. Yes, but not in the last year

´3. Yes, during the last year

**Question 3.**

On a scale of 1-10, how willing are you now to change your alcohol consumption? (1=very little willing, 10 = very willing)

**Question 4.**

On scale of 1-10, how would you rate your confidence in your ability to change your alcohol consumption?

(1= confidence in your own ability is weak, 10= confidence in your own ability is very strong)

**Question 5. We kindly ask you to fill out the attached EQ-5D-3L questionnaire [39]**


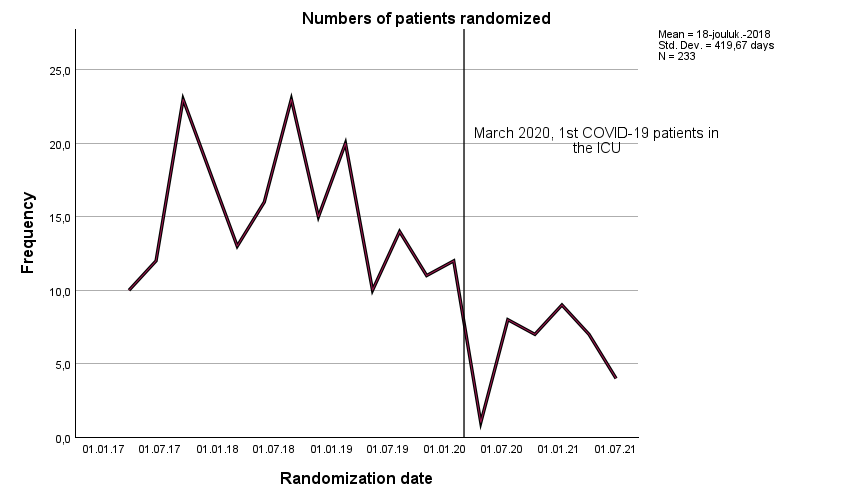


Figure E1. Rate of recruitment. The first patients was randomized in Helsinki ICU at 31. March 2017, Tampere 13. August 2017 and Turku 7. May, 2018. Pandemic slowed down recruitment considerably.

Table E1. Loss of follow-up information. Some of the follow-up information was returned but was filled incompletely.

| Missing follow-up information | BI | TAU |
| --- | --- | --- |
| 6 months | | |
| All follow-up data, n (%) | 28 (26) | 19 (18) |
| Follow-up data of alcohol consumption, n (%) | 1 (1) |  |
| Follow-up data of AUDIT score, n (%) | 0 (0) | 2 (2%) |
| 12 months | | |
| All follow-up data, n (%) | 30 (29) | 32 (32) |
| Follow-up data of alcohol consumption, n (%) | 2 (2) | 1 (1) |
| Follow-up data of AUDIT score, n (%) | 1 (1) |  |

Categorical data are presented as absolute numbers and percentages**.** AUDIT, Alcohol use disorder identification test

Table E2. ICU admission diagnosis categories

| **Diagnosis**  **category** | **BI** | **TAU** |
| --- | --- | --- |
| Sepsis/ septic shock, n=11 | 4 (3.4) | 7 (6.0) |
| Cardiac arrest, n=10 | 5 (4.3) | 5 (4.3) |
| Respiratory failure, n=27 | 22 (18.8) | 20 (17.1) |
| Cerebral hemorrhage, n=17 | 11 (9.4) | 6 (5.1) |
| Acute pancreatitis, n=42 | 22 (18.8) | 20 (17.1) |
| Acute liver failure, n= 9 | 5 (4.3) | 4 (3.4) |
| Gastrointestinal tract bleeding, n=9 | 2 (1.7) | 7 (6.0) |
| Other gastroenterology, n=7 | 2 (1.7) | 5 (4.3) |
| Traumatology, n=10 | 5 (4.3) | 5 (4.3) |
| Circulatory failure n=24 | 15 (12.8) | 9 (7.7) |
| Acid-base balance disorders, n=20 | 10 (8.5) | 10 (8.5) |
| Other, n=48 | 24 (20.5) | 24 (20.5) |

Figure E2A. Alcohol intake during preceding week in grams of pure ethanol according to sex. Comparison between patients randomized to BI and usual care at 6-month follow-up.


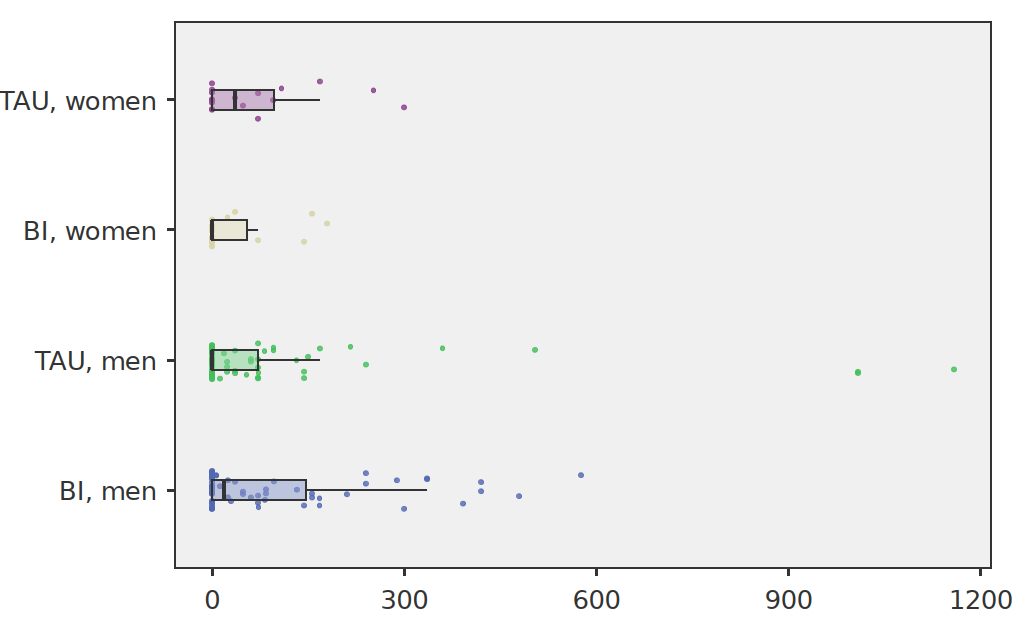


Figure E2B. Alcohol intake during preceding week in grams of pure ethanol according to sex. Comparison between patients randomized to BI and usual care at 12- month follow-up.


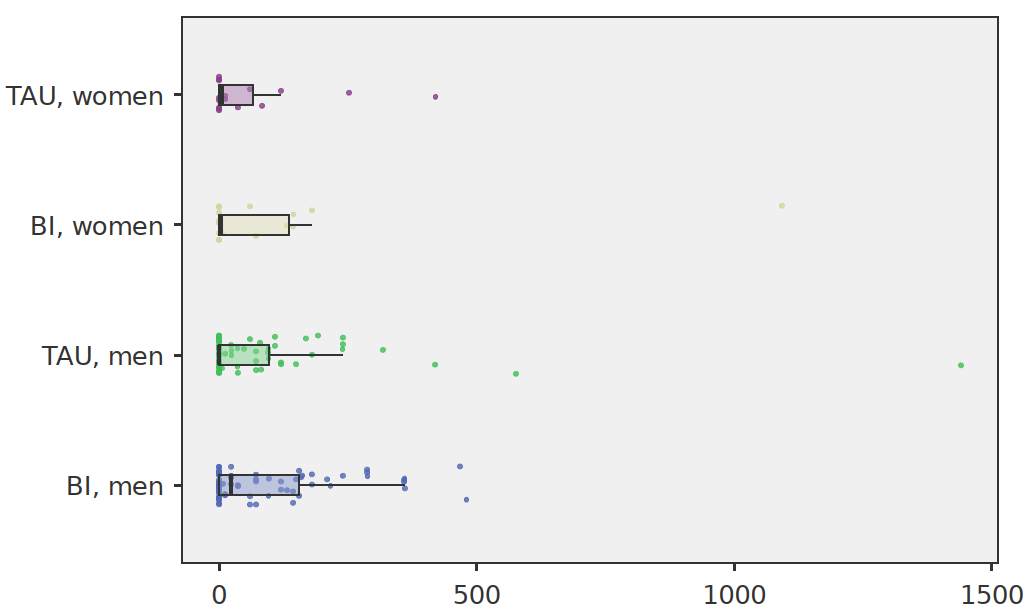


Table E3. Per protocol analysis: comparisons of the amount of alcohol consumption, AUDIT scores and change in AUDIT scores 6- and 12 months after randomization.

|  | BI | TAU | p-value |
| --- | --- | --- | --- |
| Baseline AUDIT, median (IQR) | 17 (11 – 23)  n=110 | 19 (12 – 24)  n=117 | 0.445 |
| 6 months | | | |
| Alcohol consumption (g), median (IQR) | 6.5 (0 – 123)  n=76 | 0 (0 – 72)  n=85 | 0.597 |
| AUDIT-C score, median (IQR) | 6 (4-9)  n=102 | 6 (2-8)  n=104 | 0.444 |
| ΔAUDIT-C score, median (IQR) | -2 (-4 – 0)  n=102 | -2 (-6 – 0)  n=104 | 0.133 |
| Abstinent, AUDIT score=0, n (%) | 4 (4)  n=110 | 13 (11)  n=117 | 0.032* |
| 12 months | | | |
| Alcohol consumption (g), median (IQR) | 24 (0 – 144)  n=81 | 0 (0– 96)  n=83 | 0.175 |
| AUDIT score, median (IQR) | 8 (4 – 14)  n=79 | 6 (0 – 10)  n=82 | 0.058 |
| AUDIT-C, median (IQR) | 5 (2-8)  n=79 | 4 (0-7)  n=82 | 0.234 |
| ΔAUDIT-C score, median (IQR) | -3 (-5 – -1)  n=79 | -4 (-7 – -1)  n=82 | 0.189 |
| Abstinent: AUDIT score=0, n (%) | 12 (11)  n=110 | 17 (15)  n=117 | 0.276 |

Categorical data are presented as percentages and absolute numbers (n), Continuous variables are expressed as medians and interquartile ranges [IQR]. Alcohol consumption is presented as grams of pure alcohol. AUDIT, Alcohol use disorder identification test. ΔAUDIT is change in AUDIT scores from baseline score to follow-up scores.

Table E4**.** AUDIT-scores and subdomains at baseline, and at 6- and 12-month follow-up.

|  | BI, n=117 | TAU, n=117 |
| --- | --- | --- |
| Baseline total AUDIT, median (IQR) | 18 (12 – 23)  n=117 | 19 (12– 24)  n=117 |
| Baseline AUDIT (questions 1- 3)  median (IQR) | 8 (7-10)  n=117 | 9 (7-11)  n=117 |
| Baseline AUDIT (questions 4-8)  median (IQR) | 5 (2-9)  n=117 | 5 (2-9)  n=117 |
| Baseline AUDIT (questions 9,10)  median (IQR) | 4 (2-6)  n=117 | 4 (2-6)  n=117 |
| 6-months follow-up | | |
| Total AUDIT score, median (IQR) | 14 (7 – 20)  n=109 | 10 (5– 19)  n=104 |
| AUDIT (questions 1- 3)  median (IQR) | 6 (4-9)  n=109 | 6 (2-8)  n=104 |
| AUDIT (questions 4-8)  median (IQR) | 3 (1-7)  n=109 | 2 (0-7)  n=104 |
| AUDIT (questions 9,10)  median (IQR) | 2 (0-5)  n=109 | 1 (0-4)  n=104 |
| 12-months follow-up | | |
| AUDIT (questions 1- 3)  median (IQR) | 5 (2-8)  n=84 | 4 (0-7)  n=82 |
| AUDIT (questions 4-8)  median (IQR) | 1 (0-5)  n=84 | 0 (0-3)  n=82 |
| AUDIT (questions 9,10)  median (IQR) | 2 (0-4)  n=84 | 0 (0-4)  n=82 |

Continuous variables are expressed as medians and interquartile ranges (IQR). AUDIT, Alcohol use disorder identification test.

Table E5. Willingness to change alcohol use habits (scale 1-10) and confidence in ability to change (scale 1-10) at baseline, and 6- and 12-month follow-up after ICU admission.

|  | BI  n= 117 | TAU  n=117 | p-value |
| --- | --- | --- | --- |
| Baseline |  |  |  |
| Willingness, mean, (SD) | 7 (2.8)  n=108 | n.a. |  |
| Confidence, mean, (SD) | 8 (2.4)  n=107 | n.a. |  |
| 6 months |  |  |  |
| Willingness, mean, (SD) | 7 (3.2)  n=75 | 7 (3.7)  n=80 | 0.827 |
| Confidence, mean, (SD) | 8 (1.9)  n=75 | 8 (2.4)  n=81 | 0.423 |
| 12 months |  |  |  |
| Willingness, mean, (SD) | 6 (3.7)  n=66 | 6 (3.5)  n=57 | 0.571 |
| Confidence, mean, (SD) | 8 (2.8)  n=68 | 8 (2.7)  n=59 | 0.453 |

Variables are expressed as means and standard deviations, on to scale of 0-10. N represents the number of data available for analysis. N.a.= not applicable.

Table E6. Reported EQ-5D-3L levels 12 months after ICU admission.

| Level | Mobility  n (%) | | Self-care  n (%) | | Usual activities  n (%) | | Pain/discomfort  n (%) | | Anxiety/depression  n (%) | |
| --- | --- | --- | --- | --- | --- | --- | --- | --- | --- | --- |
|  | BI | TAU | BI | TAU | BI | TAU | BI | TAU | BI | TAU |
| 1 | 42  (64.4) | 37  (59.7) | 59 (90.8) | 48 (77.4) | 50 (76.9) | 40  (64.5) | 26 (40.0) | 23 (36.5) | 38  (58.5) | 37  (59.7) |
| 2 | 23  (35.4) | 21  (33.9) | 6  (9.2) | 14  (22.6) | 12  (18.5) | 18  (29.0) | 34 (52.3) | 34  (54.0) | 26 (40.0) | 23  (37.1) |
| 3 | 0 | 4  (6.5%) | 0 | 0 | 3  (4.6) | 4  (6.5) | 5  (7.7) | 5  (7.9) | 1  (1.5) | 2  (3.2) |
| p-value | 0.114 | | 0.039* | | 0.304 | | 0.764 | | 0.795 | |

EQ-5D-3L is a standardized measure that describe health status. Level 1 indicates no problem, Level 2 indicates some problems, Level 3 indicates extreme problems. BI; brief intervention group; available data n=65/117, C; Control group, available data n=62/117.


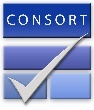
CONSORT 2010 checklist of information to include when reporting a randomised trial*

| **Section/Topic** | **Item No** | **Checklist item** | **Reported on page No** |
| --- | --- | --- | --- |
| **Title and abstract** | | | |
|  | 1a | Identification as a randomised trial in the title | 1 |
|  | 1b | Structured summary of trial design, methods, results, and conclusions (for specific guidance see CONSORT for abstracts) | 2-3 |
| **Introduction** | | | |
| Background and objectives | 2a | Scientific background and explanation of rationale | 3-4 |
|  | 2b | Specific objectives or hypotheses | 4 |
| **Methods** | | | |
| Trial design | 3a | Description of trial design (such as parallel, factorial) including allocation ratio | 4 |
|  | 3b | Important changes to methods after trial commencement (such as eligibility criteria), with reasons | 4-5 |
| Participants | 4a | Eligibility criteria for participants | 4-5 |
|  | 4b | Settings and locations where the data were collected | 4 |
| Interventions | 5 | The interventions for each group with sufficient details to allow replication, including how and when they were actually administered | 5-6 |
| Outcomes | 6a | Completely defined pre-specified primary and secondary outcome measures, including how and when they were assessed | 6 |
|  | 6b | Any changes to trial outcomes after the trial commenced, with reasons | - |
| Sample size | 7a | How sample size was determined | 7 |
|  | 7b | When applicable, explanation of any interim analyses and stopping guidelines | 7 |
| Randomisation: |  |  |  |
| Sequence generation | 8a | Method used to generate the random allocation sequence | 7-8 |
|  | 8b | Type of randomisation; details of any restriction (such as blocking and block size) | 7-8 |
| Allocation concealment mechanism | 9 | Mechanism used to implement the random allocation sequence (such as sequentially numbered containers), describing any steps taken to conceal the sequence until interventions were assigned | 4 |
| Implementation | 10 | Who generated the random allocation sequence, who enrolled participants, and who assigned participants to interventions | 4-8 |
| Blinding | 11a | If done, who was blinded after assignment to interventions (for example, participants, care providers, those assessing outcomes) and how | 8 |
|  | 11b | If relevant, description of the similarity of interventions | - |
| Statistical methods | 12a | Statistical methods used to compare groups for primary and secondary outcomes | 8 |
|  | 12b | Methods for additional analyses, such as subgroup analyses and adjusted analyses | 8 |
| **Results** | | | |
| Participant flow (a diagram is strongly recommended) | 13a | For each group, the numbers of participants who were randomly assigned, received intended treatment, and were analysed for the primary outcome | 9-10 |
|  | 13b | For each group, losses and exclusions after randomisation, together with reasons | 9 + Additional File |
| Recruitment | 14a | Dates defining the periods of recruitment and follow-up | 4 + Additional File |
|  | 14b | Why the trial ended or was stopped | 7+  Addition File |
| Baseline data | 15 | A table showing baseline demographic and clinical characteristics for each group | 11 |
| Numbers analysed | 16 | For each group, number of participants (denominator) included in each analysis and whether the analysis was by original assigned groups | 9-14  Additional File |
| Outcomes and estimation | 17a | For each primary and secondary outcome, results for each group, and the estimated effect size and its precision (such as 95% confidence interval) | 9-14  Additional File |
|  | 17b | For binary outcomes, presentation of both absolute and relative effect sizes is recommended | - |
| Ancillary analyses | 18 | Results of any other analyses performed, including subgroup analyses and adjusted analyses, distinguishing pre-specified from exploratory | 13-14 + Additional File |
| Harms | 19 | All important harms or unintended effects in each group (for specific guidance see CONSORT for harms) | - |
| **Discussion** | | | |
| Limitations | 20 | Trial limitations, addressing sources of potential bias, imprecision, and, if relevant, multiplicity of analyses | 16-17 |
| Generalisability | 21 | Generalisability (external validity, applicability) of the trial findings | 16 |
| Interpretation | 22 | Interpretation consistent with results, balancing benefits and harms, and considering other relevant evidence | 16-18 |
| **Other information** | | |  |
| Registration | 23 | Registration number and name of trial registry | 4 |
| Protocol | 24 | Where the full trial protocol can be accessed, if available | 4 |
| Funding | 25 | Sources of funding and other support (such as supply of drugs), role of funders | 18 |

Citation: Schulz KF, Altman DG, Moher D, for the CONSORT Group. CONSORT 2010 Statement: updated guidelines for reporting parallel group randomised trials. BMC Medicine. 2010;8:18.
© 2010 Schulz et al. This is an Open Access article distributed under the terms of the Creative Commons Attribution License (<http://creativecommons.org/licenses/by/2.0>), which permits unrestricted use, distribution, and reproduction in any medium, provided the original work is properly cited.

*We strongly recommend reading this statement in conjunction with the CONSORT 2010 Explanation and Elaboration for important clarifications on all the items. If relevant, we also recommend reading CONSORT extensions for cluster randomised trials, non-inferiority and equivalence trials, non-pharmacological treatments, herbal interventions, and pragmatic trials. Additional extensions are forthcoming: for those and for up-to-date references relevant to this checklist, see [www.consort-statement.org](http://www.consort-statement.org).
